# Detection of *Fusarium oxysporum* f.sp. *lactucae* race 1 and 4 via race-specific real-time PCR and target enrichment

**DOI:** 10.3389/fpls.2023.1272136

**Published:** 2023-11-23

**Authors:** Hanna Mestdagh, Kris Van Poucke, Annelies Haegeman, Tinne Dockx, Isabel Vandevelde, Ellen Dendauw, An Decombel, Monica Höfte, Kurt Heungens

**Affiliations:** ^1^ Flanders Research Institute for Agriculture, Fisheries and Food (ILVO), Plant Sciences Unit, Merelbeke, Belgium; ^2^ Department of Plants and Crops, Faculty of Bioscience Engineering, Ghent University, Gent, Belgium; ^3^ Research Station for Vegetable Production (PSKW), Sint-Katelijne-Waver, Belgium; ^4^ Vegetable Research Centre (PCG), Kruishoutem, Belgium; ^5^ Inagro, Rumbeke-Beitem, Belgium

**Keywords:** Fusarium wilt, *Lactuca sativa* L., genotyping-by-sequencing, real-time PCR, soilborne pathogen

## Abstract

*Fusarium oxysporum* f.sp. *lactucae* (Fol) causes a vascular disease in lettuce that results in significant yield losses. Race-specific and sensitive real-time PCR assays were developed for Fol races 1 and 4, which are prevalent in Europe. Using genotyping-by-sequencing, unique DNA loci specific to each race were identified and subsequently used for the design of primers and hydrolysis probes. Two assays per race were developed to ensure specificity. The two assays of each race could be run in duplex format, while still giving a sensitivity of 100 fg genomic DNA for all assays. Sample preparation methods were developed for plant tissue, soil, and surfaces, with an extra enrichment step when additional sensitivity was required. By controlling the incubation conditions during the enrichment step, the real-time PCR signal could be matched to the number of spore equivalents in the original sample. When enriching naturally infested soil, down to six conidiospore equivalents L^-1^ soil could be detected. As enrichment ensures sensitive detection and focuses on living Fol propagules, it facilitates the evaluation of control measures. The developed detection methods for soil and surfaces were applied to samples from commercial lettuce farms and confirmed the prevalence of Fol race 4 in Belgium. Monitoring of soil disinfestation events revealed that despite a dramatic decrease in quantity, the pathogen could still be detected either immediately after sheet steaming or after harvesting the first new crop. The detection method for plant tissue was successfully used to quantify Fol in lettuce inoculated with race 1, race 4 or a combination of both. Under the temperature conditions used, race 4 was more aggressive than race 1, as reflected in larger amounts of DNA of race 4 detected in the roots. These newly developed assays are a promising tool for epidemiological research as well as for the evaluation of control measures.

## 1 Introduction

*Fusarium oxysporum* f.sp. *lactucae* (Fol) is a soilborne fungal pathogen that causes vascular wilt in lettuce (*Lactuca sativa* L.), one of the most economically important leafy vegetables in Europe. The pathogen, first reported in Japan (Matuo and Motohashi, 1967) is divided into four races, of which race 1 is the most widespread. In Europe, race 1 was first discovered in 2001 in Italy (Garibaldi et al., 2002), then in Portugal (Pasquali et al., 2007), France (Gilardi et al., 2017b), Spain (Guerrero et al., 2020), Norway (Herrero et al., 2021), Belgium (Claerbout et al., 2023), Greece (Tziros and Karaoglanidis, 2023) and Ireland (van Amsterdam et al., 2023). Since 2015, a new race (race 4) has emerged as a serious threat to lettuce production in several European countries. Fol race 4 was first identified in the Netherlands (Gilardi et al., 2017). Since then, its presence has been documented in Belgium (Claerbout et al., 2018), Ireland and England (Taylor et al., 2019), Italy (Gilardi et al., 2019), and Spain (Gálvez et al., 2023). Race 2 has only been reported in Japan and race 3 in only Taiwan and Japan (Fujinaga et al., 2001; Fujinaga et al., 2003; Lin et al., 2014).

Fusarium wilt is a big problem for soil-grown lettuce. Infestation can cause significant yield losses and reduce the quality of the lettuce heads. The disease is characterized by vascular discoloration, chlorosis of the leaves, stunted growth, and wilting of the plant. The fungus can survive in the soil for several years in the form of chlamydospores carried along with crop residues (Scott et al., 2012). The persistence of Fol in soil poses significant management challenges as restrictions on the use of soil fumigants make it difficult to reduce the Fol inoculum in soil (Gullino et al., 2021). The pathogen can spread via infested soil on equipment as well as via infected seed (Garibaldi et al., 2004a; Claerbout et al., 2023). The use of resistant lettuce cultivars can be an effective control method against the disease, but currently no commercially viable resistant lettuce varieties are available.

Before the emergence of Fol, high intensity soil-based lettuce production in greenhouses was the most common cultivation method in Belgium, yielding up to five crops annually. The dramatic impact of Fol has led to research into the diversity and biology of the pathogen. Between 2015 and 2018, 78 *Fusarium* isolates were collected from symptomatic butterhead lettuce plants in Belgian commercial greenhouses (Claerbout et al., 2023). Using genotyping as well as inoculation experiments on differential cultivars, it was determined that 91% of the isolates belonged to race 4 while only 6% of the isolates belonged to race 1. The prevalence of race 4 in Belgium can be explained by the higher virulence of race 4 at soil temperatures below 18°C (Gilardi et al., 2021) which are typical for northwest Europe.

Monitoring the pathogen requires race-specific and sensitive detection techniques. Traditional methods for Fol detection are time-consuming, laborious, and may lead to false-negative results. Therefore, the development of a reliable and sensitive molecular detection method for Fol races 1 and 4 is needed to assess the prevalence and distribution of these races in lettuce production fields as well as to evaluate potential control methods. Existing detection assays for *Fusarium oxysporum* f.sp. *lactucae* races 1 and 4 include a LAMP assay (Franco Ortega et al., 2018) but this does not discriminate between the two races and it is only semi-quantitative. Conventional PCR assays have suboptimal specificity and sensitivity (Gilardi et al., 2017a; Pasquali et al., 2007). Recently, a real-time PCR assay for Fol race 1 has been developed, but not for race 4 (Sanna et al., 2022).

In this study, we aimed to develop sensitive and specific molecular detection methods for Fol race 1 and race 4 and to apply these techniques to a variety of samples such as soil, surfaces of equipment and containers, and plant tissue. The first objective was to use genotyping-by-sequencing (GBS) to identify candidate loci for race-specific primers and hydrolysis probes and to test the resulting qPCR assays for specificity and sensitivity. GBS is a high-throughput sequencing technique that can be used to distinguish the closely related race 1 and race 4, as these races cannot be discriminated based on the sequence of commonly used barcoding loci such as the intergenic spacer region (IGS) and the translation elongation factor 1-α (*tef1*) (Gilardi et al., 2017a). GBS reduces genome complexity with the use of restriction enzymes and can identify approximately 10 000 DNA loci in *Fusarium* species using a double digest with *Pst*I and *Hpa*II (Van Poucke et al., 2021; Claerbout et al., 2023). The second objective was to optimize sample processing in order to increase sensitivity. The third objective was to validate the detection method on plant tissue, on greenhouse soil, and on equipment surfaces by collecting samples at commercial lettuce farms and analyzing them using these optimized methods.

## 2 Materials and methods

### 2.1 Fungal isolates and DNA extraction

Isolates used in this study and their origin are listed in **Table 1**. The *Fusarium* cultures were stored at −80°C in 50% glycerol. To obtain genomic DNA, the isolates were grown in potato dextrose broth (Becton Dickinson) during one week at room temperature. The mycelium was dried on sterile filter paper and collected in microcentrifuge tubes. The samples were crushed to a fine powder using liquid nitrogen, sterile stainless steel beads (5 mm diameter) and a mixer mill (MM 400, Retsch). Genomic DNA was extracted using the Nucleospin Plant II kit (Macherey-Nagel) with PL1 as extraction buffer and eluted in 50 µl TE buffer. DNA concentrations were measured using the Quantus Fluorometer (Promega). DNA samples were stored at -20°C.

**Table 1:** Details of isolates used in this study.

Species (and race)	Isolate code	Host or substrate	Geographic origin	Source
*Fusarium oxysporum* f.sp.				
*lactucae* race 1	Fus1.39	*Lactuca sativa*	Belgium	(Claerbout et al., 2023)
* lactucae* race 1	Fus1.59	*Lactuca sativa*	Belgium	(Claerbout et al., 2023)
* lactucae* race 1	Fus1.60	*Lactuca sativa*	Belgium	(Claerbout et al., 2023)
* lactucae* race 1	Fus6.01	*Lactuca sativa*	Japan	(Claerbout et al., 2023)
* lactucae* race 1	244120	*Lactuca sativa*	Japan	NARO Genebank
* lactucae* race 4	Fus1.01	*Lactuca sativa*	Belgium	(Claerbout et al., 2023)
* lactucae* race 4	Fus1.02	*Lactuca sativa*	Belgium	(Claerbout et al., 2023)
* lactucae* race 4	Fus1.33	*Lactuca sativa*	Belgium	(Claerbout et al., 2023)
* lactucae* race 4	Fus1.34	*Lactuca sativa*	Belgium	(Claerbout et al., 2023)
* lactucae* race 4	Fus1.56	*Lactuca sativa*	Belgium	(Claerbout et al., 2023)
* lactucae* race 4	Fus1.58	*Lactuca sativa*	Belgium	(Claerbout et al., 2023)
* lactucae* race 4	Fus1.62	*Lactuca sativa*	Belgium	(Claerbout et al., 2023)
* lactucae* race 2	244121	*Lactuca sativa*	Japan	NARO Genebank
* lactucae* race 3	244122	*Lactuca sativa*	Japan	NARO Genebank
* lactucae* race 3	744085	naturally infested soil	Japan	NARO Genebank
* lactucae* race 3	744086	naturally infested soil	Japan	NARO Genebank
* asparagi*	CBS 143081	*Asparagus*	the Netherlands	Westerdijk Institute
* cepea*	CBS 148.25	*Allium cepa*	unknown	Westerdijk Institute
* conglutinans*	CBS 186.53	*Brassica oleracea*	USA	Westerdijk Institute
* lilli*	CBS 130322	*Lilium*	USA	Westerdijk Institute
* melonis*	CBS 420.90	*Cucumis melo*	Israel	Westerdijk Institute
* opuntiarum*	CBS 743.79	*Zygocactus truncatus*	Germany	Westerdijk Institute
* phaseoli*	CBS 935.73	*Phaseolus*	USA	Westerdijk Institute
* rhois*	CBS 220.49	*Rhus typhina*	unknown	Westerdijk Institute
* tulipae*	CBS 242.59	*Tulipa*	Germany	Westerdijk Institute
* vasinfectum*	CBS 116615	*Gossipium hirsutum*	Ivory Coast	Westerdijk Institute
* cyclaminis*	DSMZ 62315	*Cyclamen*	unknown	unknown
Other species				
*Fusarium curvatum*	CBS 247.61	*Matthiola incana*	Germany	Westerdijk Institute
*Fusarium solani*	D/20/2673	*Lactuca sativa*	Belgium	ILVO
*Fusarium poae*	MY2	unknown	Belgium	ILVO
*Fusarium avenaceum*	MY3	unknown	Belgium	PCF
*Fusarium nirenbergiae*	CBS 130303	*Solanum lycopersicum*	USA	Westerdijk Institute
*Phytophthora ramorum*		unknown	unknown	ILVO
*Trichoderma asperellum*		unknown	unknown	ILVO
*Penicillium brevicompactum*	MY27	air	Belgium	ILVO
*Aspergillus westerdijkiae*	MY13	*Capsicum annuum*	Belgium	PSKW
*Plectosphaerella plurivora*	MY61	*Lactuca sativa*	Belgium	ILVO
*Botrytis cinerea*	PCF895	*Fragaria* x *ananassa*	Belgium	(Debode et al., 2013)
*Olpidium* sp.	6	*Lactuca sativa*	Belgium	ILVO
*Verticillium dahliae*	10	unknown	Belgium	ILVO
*Sclerotinia minor*	27	unknown	Belgium	ILVO
*Cladosporium halotolerans*	MY28	unknown	unknown	ILVO
*Pythium* sp.	D/20/0242A	unknown	unknown	ILVO
*Rhizoctonia solani*		unknown	unknown	ILVO

### 2.2 Inoculum production

To produce conidiospore suspensions of Fol races 1 and 4, the isolates were grown for one week at room temperature on potato dextrose agar (PDA). Sterile water (5 mL) was added to each plate and the colony was scraped with a sterile Drigalski spatula. The suspension was filtered through a 250 µm mesh filter to remove hyphal fragments. A haemocytometer was used to determine the spore concentration and the desired concentrations were obtained by diluting with sterile water. The viability of the inoculum was determined by plating dilution series onto PDA plates.

Chlamydospores were produced following the protocol of Smith and Snyder (1971), adapted as described in Claerbout et al. (2023). In brief, air-dried sandy loam soil was autoclaved on two consecutive days. The soil (200 g) was inoculated with a microconidia suspension (10 mL, 10^6^ spores mL^-1^) and incubated at 23°C in the dark for a minimum of four weeks. The lid of the jar was closed loosely to allow drying of the soil. Before use, inoculum density was checked by dilution plating of the soil on PDA.

### 2.3 Design of race-specific real-time PCR assays for *Fusarium oxysporum* f.sp. *lactucae* races 1 and 4

Primers and corresponding hydrolysis probes were designed using Oligo Analyzer based on race-specific loci identified via genotyping-by-sequencing (GBS) as conducted by Claerbout et al. (2023) following the method of Van Poucke et al. (2021). During GBS, 81 *Fusarium oxysporum* f.sp. *lactucae* isolates were sequenced as well as 10 other *formae speciales* of *F. oxysporum* and two other *Fusarium* species. Unique DNA loci specific to either race 1 or race 4 were selected from the approximately 10 000 DNA loci (30 – 300 bp) sequenced for both races, based on the absence of the loci in the other *formae speciales* of *F. oxysporum* and other *Fusarium* species that were included in the GBS run. This was done using a custom-made Perl script (see data availability statement). For each race, candidate primer pairs were manually selected for 20 of these unique loci and risk for primer dimer formation was verified with OligoAnalyzer 1.1.2. Real-time PCR was first performed using Sybr Green with the selected primer pairs. The reaction mixture (20 µl) contained 1x PowerUp SYBR Green Master Mix (Applied Biosystems, Belgium), 300 nM of both primers and 100 pg genomic DNA. Amplification was performed using a Quantstudio Real-Time PCR system (Applied Biosystems) at 95°C for 10 min, followed by 40 cycles of 95°C for 15 s and 60°C for 1 min. A melting curve stage was added by heating to 95°C, cooling to 60°C, and slowly heating to 95°C in steps of 0.1°C/s. No-template controls (NTC) for each primer set were analyzed. The best performing primer sets were selected and tested for race-specificity. Fol race 4 isolates were analyzed with the Fol race 1 assays and Fol race 1 isolates were analyzed with the Fol race 4 assays. Hydrolysis probes were developed for primer sets with sufficient specificity. For both race 1 and race 4 of Fol, two primer sets with corresponding probes were developed. For each race, the corresponding two primer sets with probes were run in duplex format, after having shown that there was no significant difference versus running them in simplex format. The probes from the two assays of the same race were labeled with a different fluorochrome. Primer and probe sequences are shown in **Table 2**.

**Table 2:** Details of the primers and probes for the new *Fusarium oxysporum* f.sp. *lactucae* race-specific qPCR assays.

Assay	Primer/probe name	Sequence (5’ to 3’)	Amplicon size (bp)
**Fol1a**	Fol1a-F	TGTACCCTGATAATCCTGGTAC	142
	Fol1a-R	AGCTTGACTCTATCGTTGTCGA	
	Fol1a-P	(6-FAM)CCAGCAGGCTGAAGGATGCTTTGTA(QSY-7)	
**Fol1b**	Fol1b-F	CAGCATTGCCCTTTCAAGTTCA	179
	Fol1b-R	CGAGCTGCTTTAGTATTGGTGT	
	Fol1b-P	(VIC)ACCAGATTGCACCGAATTCCCTCGC(QSY-7)	
**Fol4a**	Fol4a-F	ACAATGACACCATGTGAGGTAC	223
	Fol4a-R	TTGTGTACGATCATGTGGACCA	
	Fol4a-P	(6-FAM)CGACTGCATTGGCAACCTGTGAC(QSY-7)	
**Fol4b**	Fol4b-F	GACGCCTTTCAACTTCATGCTT	195
	Fol4b-R	CCTAGATGCGTTCAAATGCTCT	
	Fol4b-P	(VIC)TGCAACGCTGGGAGAACCTTGTC(QSY-7)	

Real-time PCR with the hydrolysis probes was performed in 20 µl reactions. These contained 10 µl of 2x GoTaq qPCR Master Mix (Promega), 300 nM of the two forward and reverse primers, 50 nM of the two corresponding probes and 2 µl of DNA extract. During real-time PCR optimization, other primer/probe concentrations were evaluated (300/50, 600/50, 900/50, 300/100, 600/100 and 900/100 nM), of which 300/50 nM was finally selected. Amplification was performed using a CFX96 Touch system (Bio-Rad) at 95°C for 10 min, followed by 40 cycles of 95°C for 15 s and 60°C for 1 min. In every run, a 10-fold dilution series of race 1 and race 4 genomic DNA (0.01 to 10 ng per qPCR reaction) was included to enable quantification of DNA. No-template controls and a negative control (the other race) were also included. Each sample was run in duplicate.

### 2.4 Specificity and sensitivity of the race-specific real-time PCR assays

Specificity of the real-time PCR assays was further tested using DNA from four *Fusarium oxysporum* f.sp. *lactucae* race 1 isolates, one race 2 isolate, three race 3 isolates and seven race 4 isolates. Eleven other *F. oxysporum* isolates, four other *Fusarium* spp. and 12 isolates of other fungi or oomycetes were also tested (**Table 1**). Genomic DNA was analyzed with the newly developed real-time PCR assays (100 to 250 pg genomic DNA was added). Fus1.39 and Fus1.01 were added as positive control for the race 1 and race 4 assays, respectively.

Sensitivity of the real-time PCR assays was determined via analysis of DNA dilution series. The standard curves were generated using genomic DNA extracted from mycelia of Fus1.39 (race 1) and Fus1.01 (race 4). A ten-fold dilution series (100 ng to 100 fg per qPCR reaction) of the extracted DNA was analyzed with the real-time PCR assays.

Conidiospore standard curves were developed by producing conidiospore inoculum from Fol races 1 (Fus1.39) and 4 (Fus1.01) as described above. A dilution series was made from 10^4.4^ to 10^6.9^ spores per tube. After centrifugation (18 000 g, 5 min), the supernatant was removed and DNA was extracted with the Nucleospin Plant II kit (Macherey-Nagel) with PL1 as extraction buffer and 50 µl TE elution buffer. The genomic DNA of a range of 10^3^ to 10^5.5^ conidiospores per qPCR reaction was analyzed with the real-time PCR assays.

### 2.5 Quantification of Fol in plant tissue

To analyze the quantity of Fol in plant tissue, plant parts were placed in aluminum foil bags, flash frozen in liquid nitrogen, and kept at -20°C until further processing. The plant tissue was again placed in liquid nitrogen, removed from the aluminum bag, weighed, and added to a plastic maceration bag (Bioreba). Half the weight of the plant tissue was added in mL sterile Tris-HCl buffer (100 nM, pH 8) (e.g., 0.5 mL buffer was added to 1 g root tissue). A Bioreba homogenizer (hand model) was used to crush the tissue in the maceration bags until the plant structure was no longer visible. The suspension was collected on the opposite side of the fine-meshed gauze, quantified, and a subsample (2 mL) was taken if the resulting volume was more than 2 mL. This (sub)sample was centrifuged for 5 min at 18 000 g and the supernatant was removed. The pellets were stored at -20°C until DNA extraction. The samples were crushed into a fine powder using liquid nitrogen, sterile stainless steel beads (5 mm diameter), and a mixer mill (MM 400, Retsch). DNA extraction was performed using the Nucleospin Plant II kit (Macherey-Nagel) with PL1 as extraction buffer and elution in 50 µl TE buffer. The DNA samples were stored at -20°C. Real-time PCR was performed, and the quantity of DNA in the roots was converted to conidiospore equivalents per mg root by using DNA and conidiospore standard curves.

**Figure 1:**
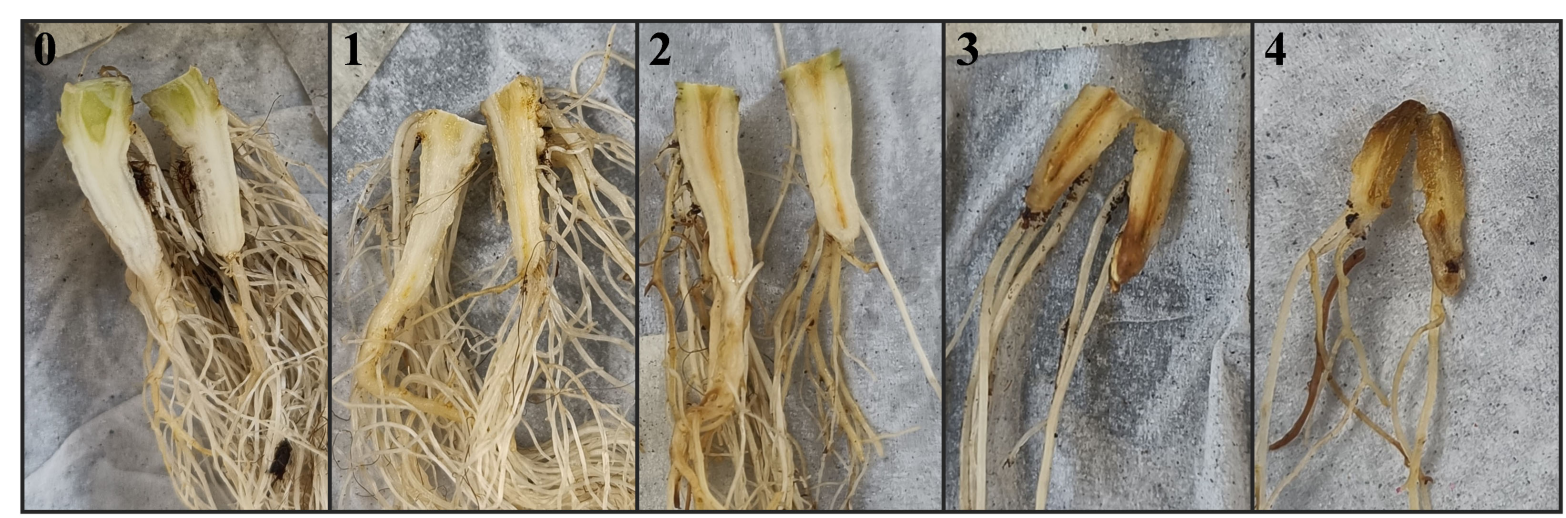
Disease scale (0-4) to score vascular discoloration of lettuce roots caused by Fol, 0 = no discoloration, 1 = light vascular discoloration, 2 = clear vascular discoloration, 3 = dark vascular discoloration, 4 = roots completely discolored and rotten.

To validate the real-time PCR assays for plant samples, we analyzed butterhead lettuce that was artificially inoculated with Fol races 1 and 4. Lettuce seeds of the cultivar ‘Cosmopolia’, which is susceptible to both Fol races 1 and 4, were surface sterilized for 30 s in 1% NaOCl and washed three times with sterile water. After sterilization, the seeds were sown in sterilized potting soil (universal type 1 structural at 56% moisture content, Snebbout N.V., Belgium) and incubated in a growth chamber at 18°C (16h/8h day/night). Sterilization of the potting soil was conducted on two consecutive days for 1 h at 121°C and 103.4 kPa. After two weeks, the lettuce seedlings were transplanted in pots with either 110 g sterile potting soil (control) or with 110 g sterile potting soil artificially infested with Fol race 1 (Fus1.39), Fol race 4 (Fus1.01), or a combination of both races. As inoculum, six-week-old chlamydospores (100 cfu g^-1^ potting soil) were used. For the pots with both races combined, 100 cfu g^−1^ potting soil of each race was added, mainly to see if there was an additive effect or whether competition occurred between the two races. Transplanted lettuce seedlings were incubated at 24°C (16h/8h day/night). Each treatment had eight replicates. After three weeks the wilting symptoms were scored on a scale from 0 to 4 following the disease scale developed by Claerbout et al. (2023). The disease index (DI) for each treatment was calculated as follows: $DI_{0-100}=\frac{\sum N_{plants}*Rating\;scale_{0-4}*100}{Total\;N_{recorded\;plants}*4}$ . The roots were washed, weighed and the vascular discoloration was scored on a scale of 0 to 4 as indicated in **Figure 1**. Disease Index (DI) was calculated for the vascular discoloration of the roots following the same equation as for the wilting symptoms. The root tissue was surface-disinfested for 1 min with ethanol (70%), and 1 min with NaOCl (1%) and subsequently washed three times with sterile, distilled water. After superficial drying of the roots, they were processed for quantification of Fol1 and Fol4 as described above. Prior to maceration in the Bioreba bags, the roots were pooled to increase the total weight. The eight roots from the treatment with Fol race 1 were pooled into two groups of four roots each based on their wilting symptoms, weight and vascular discoloration. The eight roots for the three other treatments (control, Fol race 4 and the combination of both races) were pooled into one group each.

### 2.6 Quantification of Fol in soil via target enrichment

For soil samples, an enrichment step was added to increase the sensitivity of the assays and target living propagules. This was done by incubating the samples in the semi-selective *Fusarium* medium of Komada (1975).

Greenhouse soil samples were kept at 4°C for a maximum of three days until processing. Before subsampling, the soil samples were mixed thoroughly. A soil suspension was made by diluting 100 mL of soil to 500 mL with sterile water. The suspension was thoroughly shaken before adding 10 mL to each of two 50 mL centrifuge tubes. These tubes were centrifuged (5000 g, 15 min) and supernatant was removed. The soil samples were subsequently resuspended to 10 mL with Komada medium. The samples were incubated for 48 h at 25 ˚C and 110 rpm. The tubes were set at an inclination of 20 degrees (relative to the horizontal axis). After 48 h, two subsamples of 1 mL were taken from the tubes and added to sterile 2 mL microcentrifuge tubes. These subsamples were centrifuged (18 000 g, 5 min) and the supernatant was removed. The subsamples were stored at -20°C until DNA extraction.

DNA extraction was done using the Powersoil Pro kit (Qiagen) with 50 µl elution buffer (C6). A mixer mill (MM 400, Retsch) was used for homogenization and lysis (25 Hz, twice 5 min, with inversion of the adapters in between). The DNA samples were stored at -20°C. The real-time PCR assays were used to detect and quantify Fol race 1 and race 4 in the DNA from the soil samples. We first developed a standard curve using spiked conidiospore dilution series in soil. A linear standard curve was developed that correlates the number of spores before enrichment with the DNA quantity after enrichment. By including a target DNA standard curve during real-time PCR analysis of a sample, the DNA concentration after enrichment could be calculated and this concentration was used to calculate back to the initial number of conidiospore equivalents. In addition, enrichment of soil with conidiospores was compared to enrichment of soil with chlamydospores. Soil artificially infested with chlamydospores was prepared as described above and inoculum density was checked by dilution plating of the soil on PDA. The soil was diluted with sterilized soil to a series of inoculum densities and subsequently enriched before DNA extraction and real-time PCR.

Possible interference of non-targets with the Fol race 1 and 4 assays during enrichment in soil was investigated. Chlamydospores of Fol races 1 and 4 (Fus1.39 and Fus1.01) were prepared as described above and 100 cfu of each race were added per gram of potting soil either separately or simultaneously. The three soil treatments were enriched as described above before DNA extraction and real-time PCR analysis.

### 2.7 Quantification of Fol on surfaces via target enrichment

Similar to the soil samples, an enrichment step was added for swab samples (from surfaces). Swab samples were kept at 4°C for a maximum of 3 days until processing. The swabs were transferred to or kept in a sterile 12 mL centrifuge tube after sampling and 5 mL Komada medium was added before incubation. The tubes were incubated horizontally for 48 h at 25 ˚C and 110 rpm. After 48 h the swabs were trimmed with sterile scissors and transferred to 2 mL microcentrifuge tubes with 1.5 mL of the corresponding suspension. All tubes were centrifuged (18 000 g, 5 min) and the supernatant was removed. The subsamples were stored at -20°C until DNA extraction.

DNA was extracted from the swab samples using the Nucleospin Plant II kit (Macherey-Nagel) with PL1 as extraction buffer and 50 µl TE buffer. For each swab, 200 mg zirconium beads were added with the extraction buffer and RNase. The swabs were bead-beaten at 30 Hz for 1 min using a mixer mill (400 MM, Retsch), followed by 10 min incubation at 65°C and 1400 rpm. This step was repeated three times before following the rest of the protocol from the manufacturer. The DNA samples were stored at -20°C. The real-time PCR assays were used to detect and quantify Fol race 1 and race 4 in the DNA from the swab samples. A standard curve was developed by using a spiked conidiospore dilution series on swabs. The linear standard curve correlates the number of conidiospores before enrichment with the DNA quantity after enrichment. During real-time PCR analysis of a sample, a target DNA standard curve was included, and the DNA concentration after enrichment could be calculated. This concentration was used to calculate back to the initial number of conidiospore equivalents.

### 2.8 Sampling and analysis of naturally infested soil and surfaces at lettuce farms

To determine if the real-time PCR assays can be used to quantify the pathogen in naturally infested soil and on infested surfaces, samples were obtained from commercial lettuce growers. Fol inoculum levels in the soil were monitored at two lettuce growers who performed soil disinfestation by sheet steaming. Soil samples were taken before and after disinfestation and after one subsequent crop of butterhead lettuce. Greenhouse compartments were sampled along an X-pattern with a core borer (Ø3 cm, 0-30 cm deep). The soil was mixed thoroughly before taking two subsamples for enrichment. Three compartments were sampled at farm 1 and two compartments at farm 2. At farm 2, an additional soil sample was taken with the core borer from a spot where more disease symptoms were observed. Samples were taken from the same locations on each of the three time points.

To validate the method on surfaces that may be infested with Fol, various samples were taken at five other commercial lettuce farms. Swabs were used to sample the surface of cultivators and planters. These swabs were taken from parts that came in contact with the soil. Four to five spots without any adhering soil were swabbed (25 cm² in total). Any residual soil left on the machines was also sampled, mixed and two subsamples were taken for enrichment. Greenhouse soil at these five farms was sampled as described above.

After enrichment and DNA extraction of all samples, real-time PCR analysis was performed as described above with the assays for Fol races 1 and 4. The Fol inoculum was quantified by including a DNA standard curve during real-time PCR and by using the developed standard curves that correlate the number of conidiospores before enrichment to the DNA concentration after enrichment. Numbers were then expressed in relevant units, i.e., conidiospore equivalents L^-1^ soil and conidiospore equivalents m^-2^ surface area.

### 2.9 Data analysis

Cycle threshold (Cq) values for each real-time PCR reaction were automatically determined using the CFX Maestro Software (version 5.0.021.0616) for reactions analyzed by the CFX96 Touch system (Bio-Rad). The Quantstudio Design & Analysis (version 1.5.1) was used to determine the Cq values for reactions analyzed by the Quantstudio Real-Time PCR system (Applied Biosystems). The PCR efficiency of each real-time PCR assay was calculated from the slopes of the genomic DNA standard curves as follows: $efficiency\;\left(\%\right)=\;10^{\frac{1}{slope}}-1$ .

Statistical analysis was performed with R (version 4.2.3) and R studio (version 2023.03.0) at a confidence level of α = 0.05. Ordinal data of wilting symptoms and root vascular discoloration was analyzed with the nonparametric Wilcoxon rank sum test. For the comparison of lettuce head and root weights between treatments, the distribution of the data was analyzed with Levene’s test (homogeneity of variances) and the Shapiro-Wilk test (normality). When the assumptions were fulfilled, a one-way analysis of variance (ANOVA) was performed, followed by Tukey’s multiple comparison test. When the assumptions were not met, the nonparametric Wilcoxon rank sum test was used. The ability to multiplex the real-time PCR assays and the possible interference of non-targets with Fol during enrichment in soil was analyzed with the two-samples T-test when the assumptions were met.

## 3 Results

### 3.1 Development of race-specific real-time PCR assays

PCR primers and hydrolysis probes specific to Fol race 1 and race 4 were developed. For each race, two primer sets and corresponding probes were selected to run in duplex. The four isolates of Fol race 1 (Fus1.39, Fus1.59, Fus1.60 and Fus6.01) were each amplified with the race 1 assays, while no amplification occurred with the race 4 isolates. In total, 100 pg DNA (Fus1.01) corresponded to a (average ± stdev) Cq of 27.61 ± 0.20 for assay 1a and 29.30 ± 0.27 for assay 1b. With the race 4 assays, isolates from both race 4 subgroups as mentioned in Claerbout et al. (2023) (Fus1.01, Fus1.02, Fus1.33, Fus1.34, Fus1.56, Fus1.58 and Fus1.62) were amplified. One hundred pg DNA (Fus1.39) corresponded to a (average ± stdev) Cq of 28.67 ± 0.50 for assay 4a and 27.87 ± 0.13 for assay 4b. The Fol race 4 specific assays did not amplify DNA from race 1 isolates. The Fol race 1 and race 4 assays showed no amplification of Fol race 2 and race 3 isolates, 11 F*. oxysporum* isolates from other *formae speciales*, four isolates from other *Fusarium* species and 12 isolates of other fungi and oomycetes.

**Table 3** shows the features of the standard curves generated with DNA (dilution series of 100 fg to 100 ng per qPCR reaction) from mycelium and microconidia (a range of 10^3^ to 10^5.5^ conidiospores per qPCR reaction). A reliable threshold for all assays was set at a Cq of 38. As the spore samples were analyzed in the same qPCR run as the genomic DNA, the quantity of DNA could be converted to conidiospore equivalents using the combined equations: $number\;of\;conidiospore\;equivalents=1E\left(\frac{\log\left(amount\;of\;DNA\;\left(pg\right)\right)+1.358}{0.957}\right)$ for the combined assays of race 1 and $number\;of\;conidiospore\;equivalents=1E\left(\frac{\log\left(amount\;of\;DNA\;\left(pg\right)\right)+1.463}{0.967}\right)$ for the combined assays of race 4.

**Table 3:** Standard curve features of the race-specific real time PCR assays for Fol race 1 and race 4 with DNA obtained from either mycelium or microconidia. Standard curves are in the format Cq = slope × log (X) + intercept with X either fg gDNA used per qPCR reaction or the number of conidiospores from which the extracted DNA was used in the qPCR reaction.

Description of X	Assay	Isolate	Standard curve features			Efficiency (%)	100 fg (average Cq± stdev)	csp eq at Cq=38
			slope	intercept	R²			
**gDNA from mycelium**	1a	Fus1.39 (race 1)	-3.524	34.68	0.9992	92.2	38.21±0.11	N/A
	1b	Fus1.39 (race 1)	-3.392	36.11	0.9970	97.2	38.78±0.53	N/A
	4a	Fus1.01 (race 4)	-3.458	35.53	0.9990	94.6	38.83±0.92	N/A
	4b	Fus1.01 (race 4)	-3.389	34.61	0.9965	97.3	37.18±0.72	N/A
**Microconidia**	1a	Fus1.39 (race 1)	-3.310	40.21	0.9788	100.5	N/A	0.67
	1b	Fus1.39 (race 1)	-3.157	40.10	0.9768	107.4	N/A	0.67
	4a	Fus1.01 (race 4)	-3.112	40.18	0.9666	109.6	N/A	0.70
	4b	Fus1.01 (race 4)	-3.142	40.83	0.9674	108.1	N/A	0.90

csp eq, conidiospore equivalents.

N/A, not applicable.

The ability to multiplex the two assays for each race was checked by comparing the multiplex analysis of target DNA against an analysis in which the two assays were run separately. The Fol1a and Fol1b assays detected 100.3% and 99.5% of the target (Fus1.39, 10 ng), respectively, when run in multiplex versus in singleplex format (p = 0.55 and p = 0.21). For Fol race 4 (Fus1.01, 10 ng), this was 99.0% and 99.4% for the Fol4a and Fol4b assays, respectively (p = 0.16 and p = 0.13). Similar results were obtained at 1 ng.

### 3.2 Quantification of Fol in plant samples

**Figure 2:**
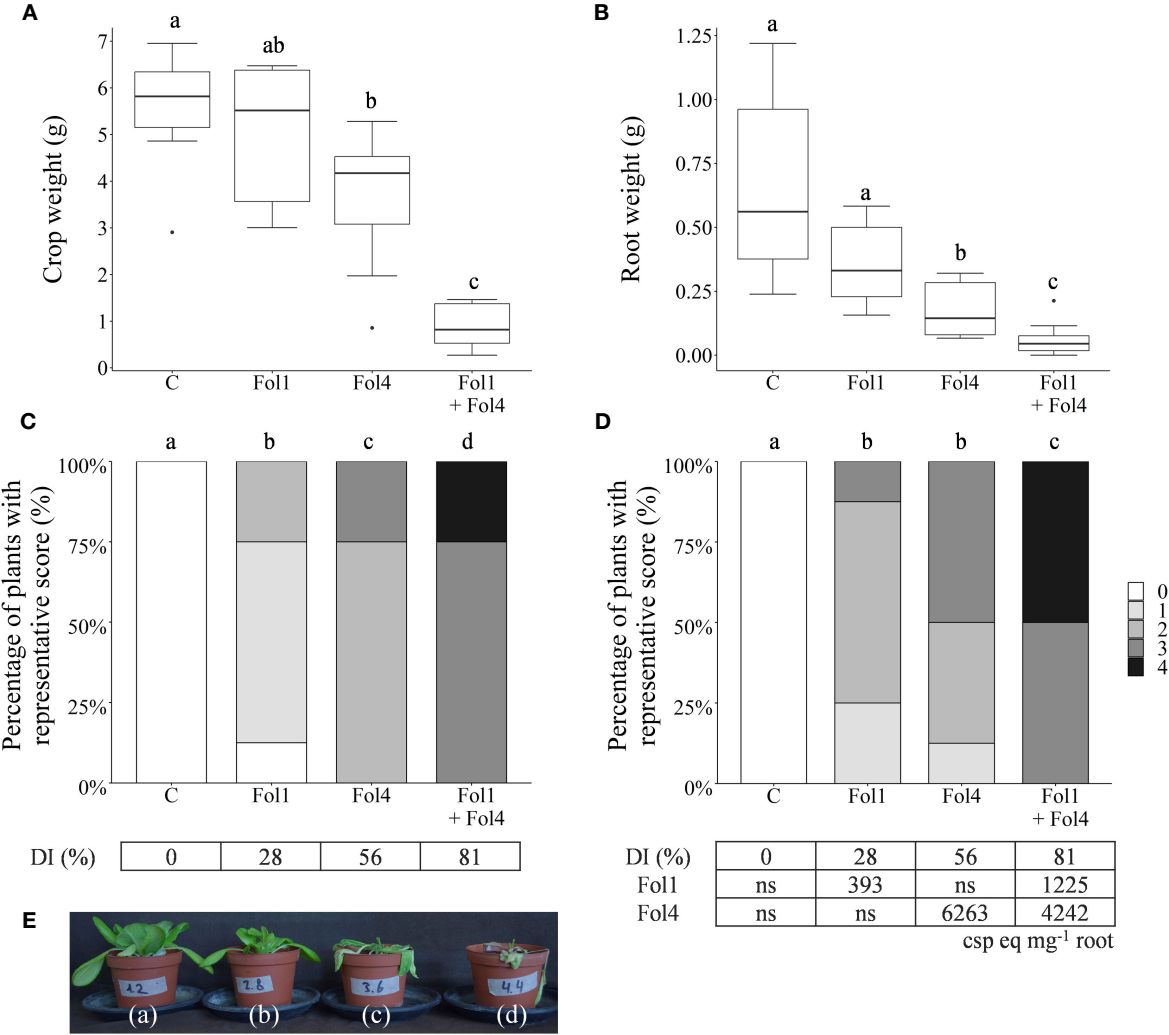
Aggressiveness of Fol race 1, Fol race 4 and the combination of both races on the susceptible butterhead lettuce cv. ‘Cosmopolia’. Inoculation was performed by adding chlamydospores (100 cfu g^-1^ soil) to sterile potting soil. For the combination treatment, 100 cfu g^−1^ soil of both races was added. Lettuce seedlings were transplanted into the inoculated soil and kept at 24°C. After three weeks the plants were analyzed by weighing the head **(A)** and root **(B)** (Tukey’s Test and Wilcoxon Rank Sum test, n = 8). Disease severity caused by Fol was assessed by scoring the wilting symptoms **(C)** and the vascular discoloration of the lettuce roots **(D)** (Wilcoxon Rank Sum test, n = 8). Conidiospore equivalents mg^-1^ root of Fol race 1 and race 4 for each treatment were quantified with real-time PCR. **(E)** Representative plants for each treatment: control (a), inoculated with Fol race 1 (b), with Fol race 4 (c) and with the combination of both races (d). Different letters in the graphs indicate statistical differences. In parts **(A)** and **(B)**, boxes represent data range (except outliers) and error bars represent the 95% confidence interval. DI, disease index; csp eq, conidiospore equivalents; ns, no signal.

Consistent with the results of Claerbout et al. (2023), Fol race 4 (Fus1.01) was more aggressive on butterhead lettuce cv. ‘Cosmopolia’ than Fol race 1 (Fus1.39) (**Figure 2**). The race 4 isolate produced more severe wilting symptoms, more vascular discoloration and a lower head and root weight. In the combination treatment, the observed additive effect in disease symptoms indicated absence of competition between the races.

The real-time PCR analysis of the roots confirmed the difference in aggressiveness between the two races, showing differences in conidiospore equivalents mg^-1^ root between the two treatments. The roots from the treatment with Fol race 1 were pooled into two groups prior to maceration. The group with lower disease severity resulted in 111 conidiospore equivalents mg^-1^ root and the other group in 674 conidiospore equivalents mg^-1^ root, resulting in a mean of 393 conidiospore equivalents mg^-1^ root. The assays allowed detection of both races in the combination treatment.

### 3.3 Quantification of Fol in soil samples

**Figure 3:**
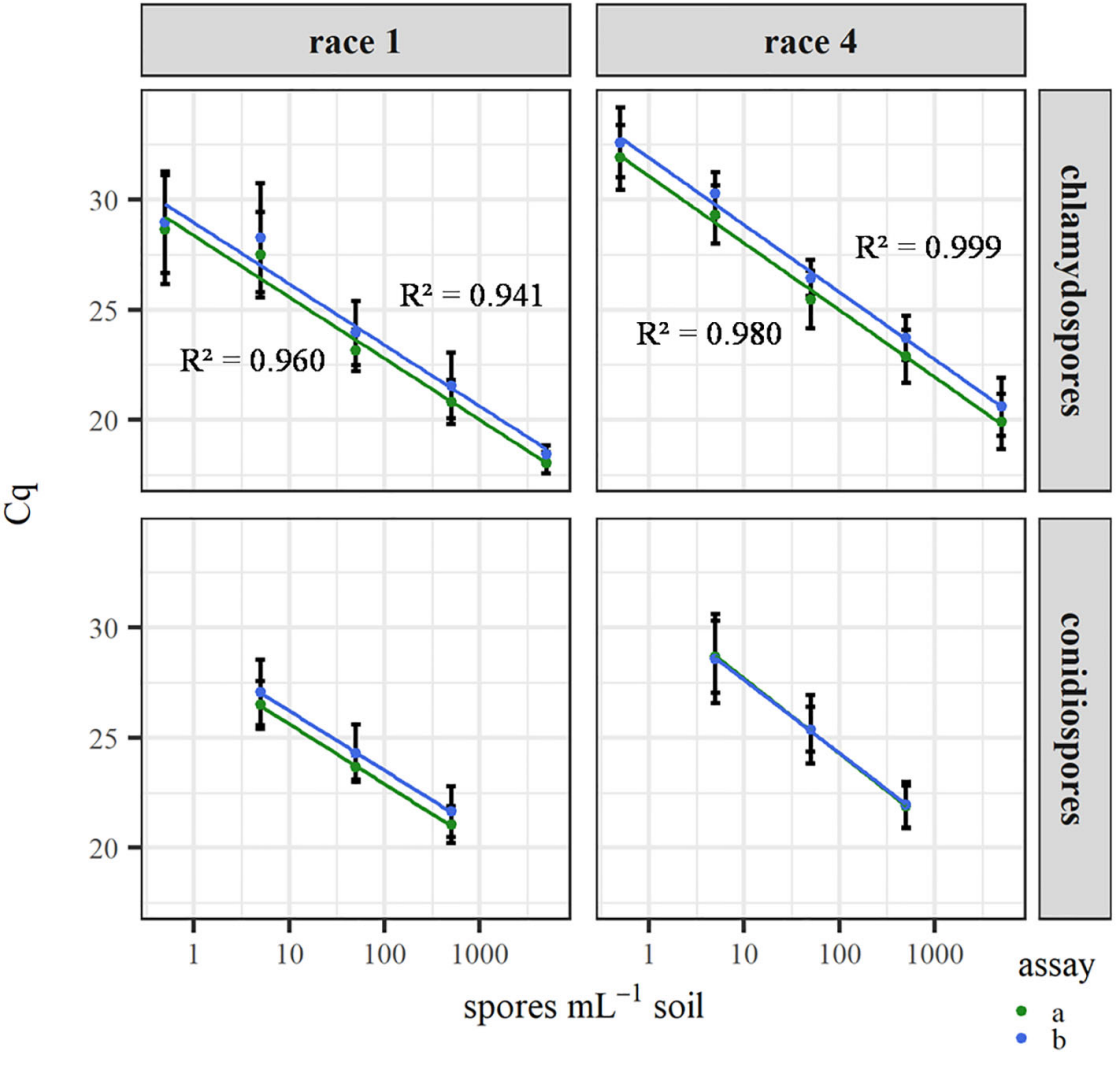
Real-time PCR quantification after enrichment of dilution series of conidiospores and chlamydospores in soil. Soil with conidiospores was prepared by inoculation with 5, 50 and 500 conidiospores mL^-1^ soil. Soil with chlamydospores was prepared as described in the text and diluted by adding sterile potting soil to obtain 0.5, 5, 50, 500 and 5000 chlamydospores mL^-1^ soil. Error bars represent standard deviation from 12, 11, 5 and 8 replicate enrichments (in soil) for Fol race 1 chlamydospores, Fol race 4 chlamydospores, Fol race 1 conidiospores and Fol race 4 conidiospores, respectively. Coefficients of determination (R²) for the conidiospore standard curves were at least 0.999 with all assays for both races.

**Figure 3** shows the standard curves that relate the inoculum densities in soil before enrichment to the Cq values after enrichment for both Fol race 1 and race 4. No differences were obtained between soils inoculated either separately or simultaneously with race 1 and 4 (p = 0.34 for Fol race 1 and p = 0.40 for Fol race 4).

To validate if the real-time PCR assays can be used for determining pathogen levels in naturally infested soil, samples were obtained from two commercial lettuce growers that performed soil disinfestation (**Table 4**). Within and between farms, large differences were observed in the number of conidiospore equivalents L^-1^ soil. At farm 1, no detection occurred in any of the soil samples after disinfestation, while detection occurred in two of the three compartments after the first crop of lettuce. One compartment at farm 2 showed a signal of Fol immediately after disinfestation, corresponding with a reduction of 88.4%. After one crop of lettuce, detection occurred in all soil samples.

**Table 4:** Real-time PCR-mediated quantification of Fol race 4 in soil samples from two commercial lettuce growers who performed soil disinfestation (sheet steaming).

Farm	Location within farm	conidiospore equivalents L^-1^ soil^a^					
		Before disinfestation		After disinfestation		After 1^st^ crop of lettuce	
		A	B	A	B	A	B
**1**	Compartment 1	1722	4716	ns	ns	7.1	ns
	Compartment 2	273	312	ns	ns	ns	ns
	Compartment 3	27	301	ns	ns	16.9	3104
**2**	Compartment 1	8748	3141	702	675	34.8	13
	Compartment 2	18 045	35 457	ns	ns	203	27
	Spot 1	51 526	49 885	ns	ns	4401	4147

ns, no signal.

a Soil samples were mixed thoroughly before taking two subsamples for enrichment (A and B).

**Table 5** shows the results of the analysis of regular soil samples and soil and swab samples taken from machinery at five other commercial lettuce farms. Soil from Farms 3 to 6 produced a Fol race 4 signal while soil from Farm 7 produced a Fol race 1 signal; the latter also corresponded with the highest number of conidiospore equivalents observed (4.8 log levels). At three farms, residual soil was found on the cultivator. All of these soil samples produced a Fol race 4 signal. The same farms had adhering soil on the planting machines with Fol race 4 detection at two of the three farms.

**Table 5:** Real-time PCR-mediated quantification of Fol in different types of soil samples and in swabs from commercial lettuce growers.

	greenhouse		cultivator			planter		
Farm	soil^c^ (csp eq L^-1^)		soil^c^ (csp eq L^-1^)		swab (csp eq m^-^²)	soil^c^ (csp eq L^-1^)		swab (csp eq m^-^²)
	A	B	A	B		A	B	
3^a^	1094	24 497	329	1114	ns	948	33	4.4
4^a^	36	5.6	19	12	1.2	ns	ns	1.2
5^a^	15 328	6057	/	/	ns	/	/	ns
6^a^	4085	3236	29 887	17 172	ns	24 469	43 402	ns
7^b^	32 086	69 300	/	/	/	/	/	/

ns, no signal; / = no sample was taken; csp eq, conidiospore equivalents.

a Detection occurred only with the Fol race 4 assays.

b Detection occurred only with the Fol race 1 assays.

c Soil samples were mixed thoroughly before taking two subsamples for enrichment (A and B).

### 3.4 Quantification of Fol in surface samples

**Figure 4:**
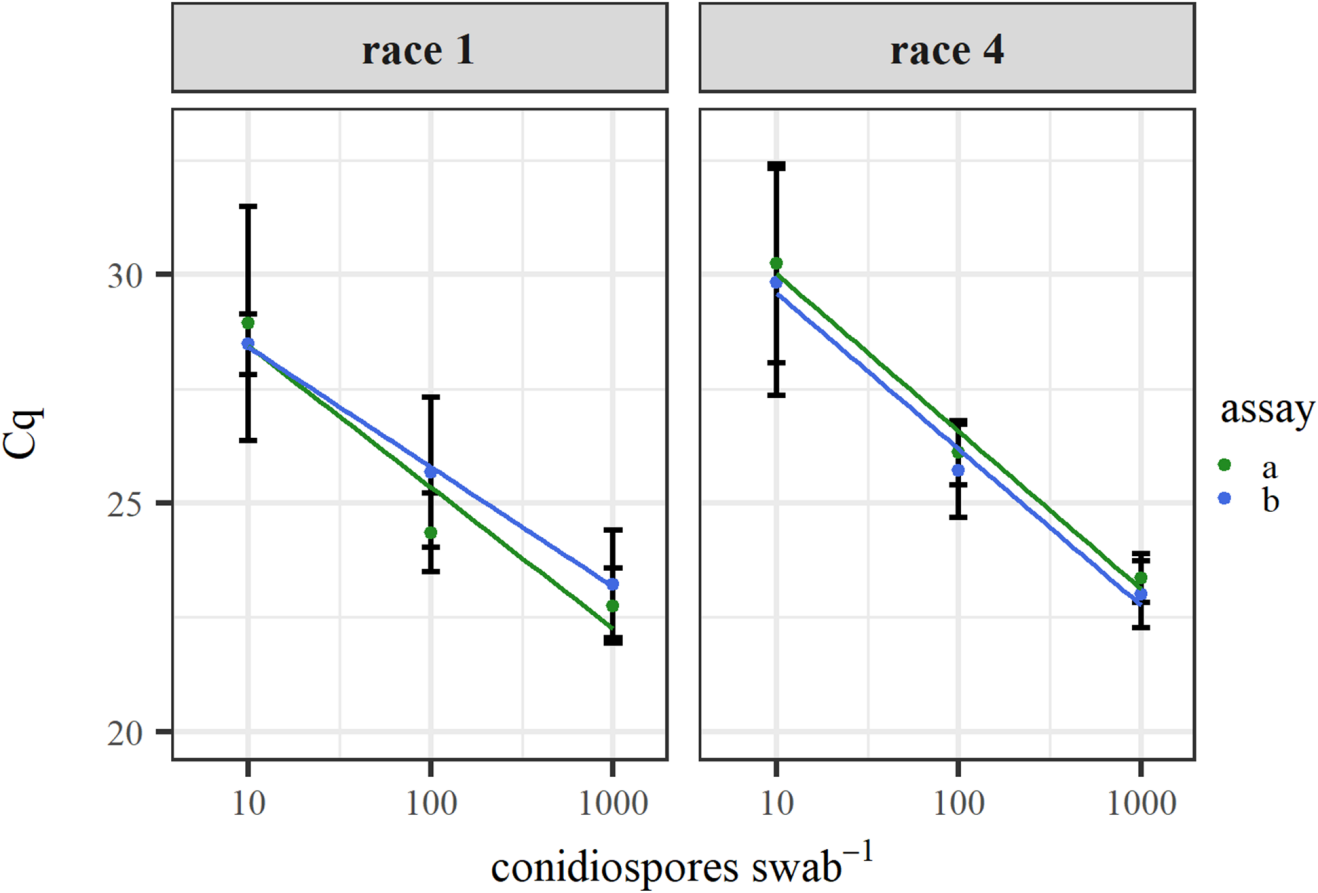
Real-time PCR mediated quantification of Fol race 1 and race 4 after enrichment of a dilution series of conidiospores spiked on swabs. Error bars represent standard deviation from five replicates.

**Figure 4** shows the quantification after enrichment of spiked conidiospores of Fol races 1 and 4 on swabs, via real-time PCR. Coefficients of determination ranged from 0.929 to 0.999. To validate this method, surface samples were taken at lettuce growers (**Table 5**). Swabs were used to sample surfaces from the cultivator and planter at four lettuce farms. Fol race 4 was detected on one of the four sampled cultivators and on two of the four planting machines.

## 4 Discussion

Within the present study, race-specific real-time PCR assays were successfully developed for *Fusarium oxysporum* f.sp. *lactucae* race 1 and race 4. Race-specific primers and corresponding hydrolysis probes were based on loci identified with genotyping-by-sequencing. Differentiation between Fol race 1 and race 4 isolates cannot be made based on *tef1*, *cmdA* and *rpb2*, but the genetic differentiation between the two races is clear when using the higher resolution GBS technique (Claerbout et al., 2023). Here the two most suitable candidate assays for each race were combined in duplex format as a protection against the possible presence of one of the loci in an unknown non-target organism. Specificity of these real-time PCR assays was confirmed based on a representative collection of pathogens, including Fol races 2 and 3, as well as a number of other fungi that could be present in the same niche. The DNA standard curves of the developed assays showed high efficiencies and high coefficients of determination. The sensitivity of the assays (100 fg genomic DNA) is comparable to that of the real-time PCR assay for Fol race 1 from Sanna et al. (2022).

The introduction of an enrichment step in the sample preparation method results in a sensitive detection method. Without enrichment of a soil sample, the detection limit of the real-time PCR assays (100 fg) would theoretically correspond to a detection limit in the soil of approximately 6000 conidiospore equivalents L^-1^ soil, while the lowest detection in naturally infested soil in this study was six conidiospore equivalents L^-1^ soil. This demonstrated the need for the enrichment technique. Another advantage of this sample preparation method is its focus on living (or at least non-dormant) and thus potentially infective fungal propagules, which represents a better risk assessment. A critical point is the potential variation in growth between enrichments of replicate samples. Based on the results presented, the variation among replicate samples analyzed at the same time was acceptable, but care should be taken in the comparison of samples analyzed at different time points: minor deviations in the growth response of the chlamydospores, e.g. as a result of additional storage time, could create differences in spore equivalents due to activity rather than a difference in number. Depending on the research objective, more enrichment replicates could be included and attention could be paid to the simultaneous enrichment of the samples to minimize this variation. Besides soil and surface samples, the enrichment method could also be applied to lettuce seed (data not shown). High sensitivity is required in obtaining a proper detection method for Fol in seed as few contaminated seeds in a large seed lot can disseminate the pathogen (Garibaldi et al., 2004a; Gordon and Koike, 2015). Sensitive assays to detect Fol in seed already exist but are not race-specific (Mbofung and Pryor, 2010; Franco Ortega et al., 2018).

The detection and quantification of Fol race 4 in soil samples from two commercial lettuce growers who performed soil disinfestation indicate that the real-time PCR assays are a potential tool for assessment of soil inoculum levels. At one farm these inoculum levels ranged from 27 to 4716 conidiospore equivalents L^-1^ soil before disinfestation to no detection in any of the compartments after disinfestation, proving that sheet steaming can be an effective method in reducing the Fol soil population. However, after one subsequent crop of lettuce, detection occurred at two of the three compartments ranging from 7 to 3104 conidiospore equivalents L^-1^ soil, indicating that Fol was still present but was either not detected or had been reintroduced after steaming. The first hypothesis seems more likely when taking into consideration the results of the second farm, which still showed some detection in one of the two sampled compartments immediately after disinfestation. Non-detection can be the result of presence under the detection limit of the used assays or the sampling method. Soil was taken up to a depth of 30 cm, thus it is also possible the pathogen survived in deeper layers. At the second farm, a specific spot was sampled where more disease symptoms were observed. As expected, more conidiospore equivalents were detected in this soil sample relative to the overall soil sample of the two compartments. The new detection methods allowed evaluation of disinfection methods and demonstrated that the effect of sheet steaming is clearly temporary. Even low numbers of remaining pathogen in the soil will increase in the host and lead to accumulation of Fol soil inoculum levels because lettuce roots are incorporated in the soil after harvest.

Further evaluation of the race-specific real-time PCR assays was conducted by collecting soil samples from five additional commercial lettuce farms. This resulted in Fol race 4 detection at four farms and Fol race 1 detection on one farm. Additionally, soil adhering to cultivators and planters was obtained, leading to the detection of Fol in five out of the six sampled machines. This demonstrates the importance of machinery in the spread of the pathogen. Furthermore, even on swabbed surfaces devoid of any adherent soil on four cultivators and four planters, Fol was detected in three cases, albeit at low levels. These results highlight the importance of not only rinsing off soil but also implementing disinfection measures for the equipment in order to effectively mitigate the spread of the pathogen. The race-specificity of the assays allowed us to confirm that most farms seem to be affected by Fol race 4 and not Fol race 1. As mentioned before, the greater pathogenic importance and prevalence of Fol race 4 in western Europe is probably due to the lower soil temperatures during most of the production year. Knowledge of the race present at a given farm or farm compartment also has implications for the choice of cultivars, as newly released commercially viable cultivars will initially have resistance to either race 1 or race 4 but not both.

The use of the race-specific real-time PCR assays for detection of Fol in plant tissue was validated by conducting a plant experiment with both races 1 and 4. Fol race 4 was more aggressive on butterhead lettuce cv. ‘Cosmopolia’ than Fol race 1, confirming results of Claerbout et al. (2023). This also showed in the results of the real-time PCR analysis of the roots, as more conidiospore equivalents mg^−1^ root were detected in the treatment with Fol race 4. Comparing the two separate treatments where race 1 or race 4 was inoculated with the combined treatment did not result in any sign of competition between the two races.

The developed real-time PCR assays and the optimized sample preparation method can be used for risk assessment of Fol. In doing this, multiple influencing factors should be considered, such as the sampling method. This method is important because Fol is not uniformly distributed in the soil, as evidenced by spots with higher inoculum (and corresponding disease) levels. This was the case at lettuce farm 2, as higher concentrations occurred in a specific spot in the greenhouse where more disease symptoms were noted. Associating risk of disease to observed inoculum levels may also be difficult under field conditions for other reasons than the patchy distribution, as dose response is affected by several other factors such as temperature, cultivar, nutrient status and the microbiome (Garibaldi et al., 2004b; Scott et al., 2009; Okubara et al., 2013; Veresoglou et al., 2013).

Risk assessment can also be performed by using the real-time PCR assays for the detection of Fol in (semi) resistant cultivars and potential other crops for crop rotation. While these crops may not show *Fusarium* symptoms, their xylem vessels might harbor considerable amounts of Fol (Scott et al., 2014). By comparing the quantities of Fol races 1 and 4 in non-symptomatic crops to the amount of Fol in roots of susceptible lettuce cultivars, the risk of inoculum build-up can now be assessed.

## 5 Conclusions and future perspectives

This study provides sensitive and race-specific real-time PCR detection methods for Fol races 1 and 4. Race-specific targets for these assays could be identified via genotyping-by-sequencing (GBS). The combination of duplicate assays per race protects against possible future specificity issues. An enrichment method is needed for cases where added sensitivity is crucial or where the focus is on the detection of living propagules. We demonstrated the applicability of the assays in complex matrices such as root tissue and soil. The molecular detection methods and optimized sample preparation provide easy tools for race identification, which should contribute to better disease management practices and ultimately reduce the economic losses caused by this pathogen.

The assays can now be used to study the spread of the pathogen as well as to evaluate the effect of control measures such as different soil disinfestation methods and the use of new resistant varieties and alternative crops. The added sensitivity obtained with the enrichment method could be applied to examine the risk of introduction or spread of the pathogen via seeds or irrigation water. This study provides the first quantitative data on the presence of Fol in greenhouse soil. It can now be extended to an in-depth study of the two- and three dimensional distribution of the pathogen in greenhouse soils. Assays with in-field applicability, such as LAMP and RPA, could be developed based on the same race-specific loci.

## Data availability statement

The original contributions presented in the study are included in the article/supplementary material. Further inquiries can be directed to the corresponding author. More information on the GBS data processing as well as all scripts can be found on https://gitlab.ilvo.be/genomics/GBS/GBS_Fol4 or on Zenodo with DOI 10.5281/zenodo.8175922.

## Author contributions

HM: Conceptualization, Writing – review & editing, Formal Analysis, Investigation, Methodology, Validation, Visualization, Writing – original draft. KV: Investigation, Methodology, Writing – review & editing. AH: Formal Analysis, Software, Writing – review & editing. TD: Conceptualization, Project administration, Resources, Writing – review & editing. IV: Conceptualization, Funding acquisition, Project administration, Supervision, Writing – review & editing. ED: Project administration, Resources, Writing – review & editing. AD: Conceptualization, Funding acquisition, Project administration, Resources, Writing – review & editing. MH: Conceptualization, Funding acquisition, Project administration, Supervision, Writing – review & editing. KH: Conceptualization, Funding acquisition, Project administration, Supervision, Writing – review & editing.
